# Involvement of the Kinin B1 Receptor in Increased Permeability of Cerebral Microvessels in Rats Subjected to Autoimmune Encephalomyelitis

**DOI:** 10.3390/cells13191641

**Published:** 2024-10-02

**Authors:** Grzegorz Sulkowski, Beata Dąbrowska-Bouta, Małgorzata Frontczak-Baniewicz, Lidia Strużyńska

**Affiliations:** 1 Laboratory of Pathoneurochemistry, Department of Neurochemistry, Mossakowski Medical Research Institute, Polish Academy of Sciences, 5 A. Pawińskiego str., 02-106 Warsaw, Poland; gsulkowski@imdik.pan.pl (G.S.); bbouta@idik.pan.pl (B.D.-B.); 2 Electron Microscopy Research Unit, Mossakowski Medical Research Institute, Polish Academy of Sciences, 5 A. Pawińskiego str., 02-106 Warsaw, Poland; mbaniewicz@imdik.pan.pl

**Keywords:** EAE, kinins, microvessels, DALBK, claudin, occludin, astroglia, MS

## Abstract

Kinins are vasoactive peptides that are involved in various cellular mechanisms, including the inflammatory response. Kinins, released in vessel walls, exacerbate inflammation by modulating the production and release of pro-inflammatory factors via two types of G protein-related receptors—B1 and B2 receptors. B1 R is overexpressed during the inflammation that accompanies numerous neurological disorders, including multiple sclerosis (MS), in which loss of BBB integrity is an early pathomechanism of the disease. In this work, we apply pharmacological inhibition of the kinin B1 receptor with DALBK to investigate its effect on blood–brain barrier (BBB) permeability during the course of EAE, an animal model of MS. Functional, ultrastructural and molecular analyses were performed. The expression of selected BBB-associated proteins such as occludin and claudin-5 was assessed, as well as the astrocytic marker GFAP. We show that administration of a specific antagonist attenuates neurological symptoms in EAE rats and recovers the downregulation of TJ proteins and BBB leakage observed during the course of the disease, as well as significantly reducing the disease-specific activation of astroglia. The results show that B1 R-mediated signaling is involved in inducing molecular changes at the level of cerebral microvessels, leading to increased permeability of the BBB following neuroinflammation in EAE.

## 1. Introduction

Multiple sclerosis (MS) is an autoimmune inflammatory demyelinating disease, affecting young adults, that leads to progressive damage of the central nervous system (CNS). The disease exhibits both inflammatory and neurodegenerative features and is characterized by the presence of focal lesions in the white and grey matter of the spinal cord and brain. During the course of the disease, the host’s immune system becomes sensitized to its own myelin antigens. Activated peripheral immune cells infiltrate the brain and spinal cord, initiating immune-mediated processes that lead to demyelination and progressive neurodegeneration accompanied by inflammatory reaction [1]. T cells, macrophages and activated glial cells release a range of pro-inflammatory mediators and generate free radicals and cytotoxic excitatory amino acids, thereby contributing to oxidative stress, myelin damage and axonal degeneration [2]. 

Loss of BBB integrity in the early stages of MS and facilitated transvascular migration of immune cells are important pathomechanisms of the disease contributing to the development of the inflammatory cascade. Perivascular inflammatory infiltrates of T cells, B cells and monocytes are initiated around post-capillary venoles and veins and spread further into the surrounding white and grey matter [3,4]. The main diagnostic hallmark of the disease is the presence of demyelinating lesions in the CNS of MS patients, accompanied by reactive gliosis. Activated glial cells, both microglia and astroglia, amplify the inflammatory reaction by releasing proinflammatory mediators such as cytokines, chemokines and adhesion molecules of both a pro- and anti-inflammatory nature [5]. Increased levels of pro-inflammatory cytokines further affect the integrity of the BBB. 

The BBB is a complex system located in the cerebral microvessels, responsible for the restrictive transport of molecules from the blood to the CNS. Morphologically, it consists of endothelial cells connected by tight junctions, basement membrane and pericytes. These elements of the anatomical barrier, together with perivascular astrocytes, neurons and microglia form the so-called neurovascular unit (NVU), which is the functional unit of cerebral microvessels [6]. The tight junction complexes between endothelial cells are a complex of transmembrane and cytoplasmic proteins, including claudins and occludins, zonula occludens (ZO) proteins and junctional adhesion molecules (JAMs), which link these transmembrane proteins to the cytoskeleton [7]. 

During neuroinflammation, in addition to cytokines and chemokines, vasoactive peptides, known as kinins, are produced at the vessel walls [8]. These molecules are overexpressed in the injured or inflamed tissue [9,10], where they contribute to vascular leakage [10] and infiltration of immune cells [11] by modulating the production and release of pro-inflammatory mediators. 

The biological activity of kinins is mediated by two types of G protein-related receptors—the B1 and B2 receptors. The B2 receptor is constitutively expressed in the central and peripheral nervous systems, whereas B1 R is overexpressed during inflammation, infection or injury. The biological significance of B1 R activation during inflammation is relevant in the context of impaired BBB and neutrophil accumulation in inflamed tissues, as activation of these receptors is believed to lead to inflammation through the release of proinflammatory cytokines and increased vascular permeability (for a review, see [12]).

The involvement of kinins and their receptors in the pathogenesis of MS/EAE has been previously suggested. Increased levels of components of the kalikrein–kinin system have been found to increase both in the nervous tissue of MS patients [13] and in the blood of EAE rats [14]. Experimental evidence has also shown that the expression of the kinin receptor B1 increases in parallel with maximal neurological symptoms during EAE and in active lesions in human MS samples [15]. Overexpression of B1 R has been observed in the brain tissue of rats subjected to EAE starting from the early asymptomatic phase of the disease, and pharmacological inhibition of this receptor partially alleviates neurological symptoms in animals [16]. Moreover, the activity of B1 R has been shown to correlate with its expression in peripheral T lymphocytes in MS patients [17].

To further expand our knowledge on the role of B1R in EAE, in this work, we investigate the possible involvement of the kinin B1 receptor in the remodeling of the BBB in EAE-associated neuroinflammation. Pharmacological inhibition of B1 R was accomplished using a specific antagonist (des-Arg^9^-[Leu^8^]-bradykinin; DALBK). Functional, ultrastructural and molecular determinants of the BBB status were investigated.

## 2. Materials and Methods 

### 2.1. Animal Model and Experimental Design 

Eight-week-old female Lewis rats (66 in total) were provided by the animal house of the Mossakowski Medical Research Institute of the Polish Academy of Sciences (Warsaw, Poland). All procedures involving animals were carried out in accordance with the European Communities Council Directive 2010/63/EU for the Care and Use of Laboratory Animals. The ethical protocol was approved by the IV Local Experimental Animal Care and Use Committee (66/2015). 

The experiment was designed as previously described [16]. Briefly, the rats were divided into 3 groups: (1) control non-immunized; (2) EAE; (3) EAE + DALBK. To induce EAE, the rats were anesthetized with sodium pentobarbital and immunized subcutaneously in both hind feet with 100 µL of inoculum containing guinea pigs’ spinal cord homogenate in PBS (1:1 ratio) emulsified with complete Freund’s adjuvant (CFA) and 2 mg/mL of *Mycobacterium tuberculosis* H37Ra (Difco, Detroit, MI, USA).

In the DALBK-treated group, a specific antagonist of B1 R, DALBK (Sigma-Aldrich, St. Louis, MO, USA), was administered i.p. to the animals at a dose of 50 nmol/kg b.w. 1 h post-immunization (day 0) for 5 consecutive days. The dose was determined based on our previous results [16] and available reports [18,19].

The effect of DALBK on control non-immunized rats was assessed prior to the main experiment, and no obvious changes in the condition or behavior of the animals were observed in this group. Rats were weighed daily and monitored. The clinical scores of neurological symptoms of EAE were assigned according to a five-point scale [20].

Animals were sacrificed in the asymptomatic and symptomatic phases of EAE, i.e., 6 days post-immunization (d.p.i.) and 15 d.p.i., in order to collect material for analysis.

### 2.2. Determination of Blood–Brain Barrier (BBB) Integrity 

The functional assessment of the BBB integrity was performed based on a method using Evans blue (EB) dye according to the detailed protocol described by Goldim et al. [21]. Animals were anesthetized with 10% (*w*/*v*) ketamine hydrochloride and 2% (*w*/*v*) xylazine hydrochloride; 2% (*w*/*v*) EB in saline (Sigma-Aldrich, E2129) was injected intravenously to the rats and allowed to circulate for 1 h. They were then perfused intracardially with saline to remove the dye from circulation. After decapitation, brains were dissected, weighted and homogenized in 500 μL 50% TCA. Samples were then centrifuged at 10,000× *g* for 20 min, and the supernatants were diluted four-fold with ethanol. The absorbance was measured at 620/680 nm against blind sample (50% TCA). The dye concentrations in the tissue were calculated using a standard curve of EB dissolved in ethanol. 

### 2.3. TEM and Immune–TEM Analyses

For TEM analysis, the animals were anesthetized with Nembutal (80 mg/kg b.w.) and perfused through the heart, first with 0.9% NaCl in 0.01 M sodium-potassium phosphate buffer (pH 7.4) and then with fixative solution, i.e., a mixture of 2% paraformaldehyde and 2.5% glutaraldehyde in 0.1 cacodylate buffer, pH 7.4. After decapitation, brain samples were collected from the 3 groups of animals. In the EAE and EAE + DALBK groups, samples were only taken from animals 15 d.p.i. Obtained material was fixed in the above ice-cold fixative solution and post-fixed in 1% OsO4, dehydrated in an ethanol gradient and embedded in epoxy resin (Epon 812) and cut into ultrathin sections. Sections were then contrasted routinely with lead citrate and uranyl acetate and examined by TEM (JEM-1200EX, Jeol, Kanagawa, Japan). 

Immune–TEM analysis of B1 R localization at the ultrastructural level was performed in brain samples of rats obtained as described above. Prior to the embedding in epoxy resin, samples were blocked in 1% BSA in PBS buffer for 15 min and incubated with primary anti-B1R antibody overnight (Santa Cruz Biotechnology, Inc., Dallas, TX, USA; dilution 1:50). This was followed by labeling with colloidal gold–conjugated protein A (10 nm gold particles; dilution 1:50) (Sigma-Aldrich, Poznań, Poland) for 2 h and subsequent washing in PBS buffer and distilled water. The remaining steps were performed as in the routine TEM protocol. 

Ultrastructural analyses were performed in Electron Microscopy Research Unit at Mossakowski Medical Research Institute Polish Academy of Sciences (MMRI PAS), Warsaw, Poland. 

### 2.4. qRT-PCR Analysis

The animals were decapitated at appropriate time points (6 and 15 d.p.i.), and the brains were dissected, frozen in liquid nitrogen and stored at –80 °C for further analyses. Brain tissues obtained from 4 rats were divided in half and used for both qRT-PCR analysis and Western blots. Total RNA was extracted from the brains using TRI Reagent (Sigma-Aldrich, St. Louis, MO, USA). Reverse transcription of 2 µg of total RNA was performed at a final volume of 20 µL using random primers and AMV reverse transcriptase. TaqMan assays were used for quantitative real-time PCR analysis using the primers of Cldn5 (Rn 01753146_s1), Ocln (Rn00580064_m1) and Gfap (Rn01253033_m1) (Termo Fisher Scientific Inc., Waltham, MA, USA). Real-time PCR was performed on a Light Cycler 96 System (Roche Diagnostic GmbH, Mannheim, Germany). The relative expression levels of mRNAs were normalized to actin (Actb) as a reference gene and calculated using the ΔΔ Ct method.

### 2.5. Western Blot Analysis

Equal amounts of brain homogenates (50 µg protein) were loaded onto and separated on a 12% SDS-PAGE gel electrophoresis, transferred onto nitrocellulose membranes and examined for the expression of selected tight junction proteins and GFAP. Membranes were incubated with primary polyclonal antibodies: anti-claudin 5 (1:1000; Invitrogen, Waltham, MA, USA), anti-occludin (1:500; Invitrogen, Waltham, MA, USA), anti-GFAP (1:500; Merck Life Science, Poznań, Poland) and a polyclonal anti-β actin antibody (1:500; MP Biomedicals, Warsaw, Poland), which was used as an internal standard. After overnight (4 °C) incubation, secondary antibody conjugated with HRP (1:500; MP Biomedicals, Warsaw, Poland) was applied for 30 min after washing the membranes three times in PBS. Specific bands were detected with a chemiluminescence ECL kit (Amersham, Buckinghamshire, UK), scanned and quantified using ImageJ software.

### 2.6. Isolation of the Capillary Fraction

The capillary fraction was isolated from gray matter prepared from freshly dissected rat brain [22]. After homogenization in Ringer’s buffer, samples were centrifuged at 1500× *g* for 10 min, and the pellet was collected and re-suspended in the same solution. This procedure was repeated two times. The final pellet was homogenized in 10 mL of 0.25 M sucrose and centrifuged in a gradient of molar sucrose concentrations 0.25:1:1.5 M, at 30,000× *g* for 30 min. The fraction of microvessels was collected from the bottom of the tube and checked at once for purity using a light microscope (Zeiss Axiovert 25). 

### 2.7. Immunohistochemical Procedure

For immunohistochemical analysis, samples of capillary fraction were smeared on slides and fixed in 4% paraformaldehyde for 20 min to preserve their cellular structure. To allow the antibodies to penetrate the cells, slides were then permeabilized using 0.1% Triton X-100 for 30 min and stained with primary anti-occludin (1:50; Invitrogen, Waltham, MA, USA) and anti-claudin 5 (1:50; Invitrogen, Waltham, MA, USA) antibodies. Then, secondary antibody conjugated with Alexa Fluor 488 or Alexa Fluor 546 was applied. Cell nuclei were stained with Hoechst dye (Sigma-Aldrich, Poland). Immunostaining specificity was checked by omitting the primary antibodies during the incubation. The slides were examined using an LSM 780/ELYRA PS.1 super resolution confocal system (Carl Zeiss, Jena, Germany). Capillary fractions obtained from three animals in each group were used for microscopic examination. Three microscopic slides were prepared from each sample. At least nine comparable regions of interest (ROIs) were selected from each slide using software tools and analyzed. The mean fluorescence intensity of the ROIs was normalized to the total cellular fluorescence measured using Zen 2.6 software (Carl Zeiss, Jena, Germany). 

Confocal microscopy analysis was performed in Laboratory of Advanced Microscopy Techniques at MMRI PAS, Warsaw, Poland. 

### 2.8. Statistical Analysis 

The results are presented as means ± SD from the number of experiments indicated in the figure captions. Significance of the result was assessed by one-way ANOVA followed by post hoc Dunnett’s test to identify changes significantly different from the controls at p levels < 0.05. The statistical analysis of data was performed using GraphPad Prism v. 6.0 software (La Jolla, San Diego, CA, USA).

## 3. Results

### 3.1. The Influence of DALBK on the Course of the Disease

Immunized rats were monitored daily for weight loss and development of neurological deficits, which were scored using a five-point-scale according to Kerschensteiner et al. [20]. The first clinical symptoms of EAE started to develop at 10–11 d.p.i. and peaked at 14–15 d.p.i. as progressive paralysis of the tail and hind limbs and reduced physical activity. Full recovery was observed at 21–22 d.p.i. In animals developing EAE, a significant loss of body weight was noted compared to non-immunized control rats during the symptomatic phase.

Administration of DALBK did not affect the loss of body weight, but significantly delayed the onset of disease by 3 days and reduced neurological deficits by one point compared to the EAE group. The maximum disease score decreased from 4 (EAE) to 3 (EAE + DALBK) (Table 1). Blockade of B1 R by an antagonist noticeably improved the overall condition of the animals. 

### 3.2. The BBB Integrity during the Course of EAE–Functional Test and TEM Analysis

**Figure 1:**
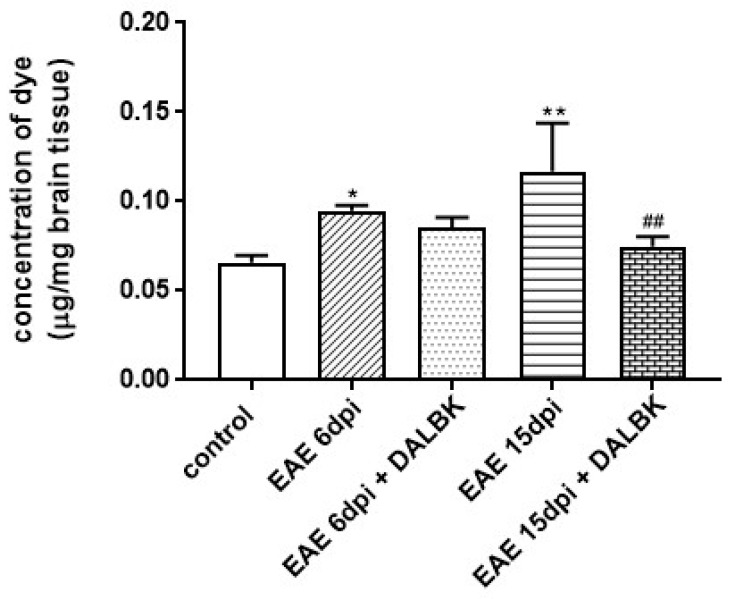
The functional test of blood–brain barrier (BBB) integrity. The graph shows the concentration of EB dye in brain homogenates obtained after perfusion of rats: (i) control; (ii) immunized; (iii) immunized administered with DALBK. Immunized (EAE) rats were sacrificed in asymptomatic (6 d.p.i) and symptomatic phases of the disease (15 d.p.i.). Results are means ± SD from 4 animals in each group; * *p* < 0.05; ** *p* < 0.01 vs. control; ^##^ *p* < 0.01 vs. EAE 15 d.p.i. (one-way ANOVA followed by post hoc Dunnett’s test).

Extravasation of EB dye is commonly used as an indicator of BBB disruption. By measuring the concentration of EB in rat brain homogenates, we quantitatively assessed the permeability of the cerebral microvessels under EAE conditions. The permeability of the BBB has already increased slightly but significantly in the asymptomatic phase of EAE (6 d.p.i.) (*p* < 0.05 vs. control) and tends to increase along with the development of the symptomatic phase of the disease (15 d.p.i.) (*p* < 0.01 vs. control). Administration of DALBK significantly decreased the concentration of EB, indicating the improvement of the BBB integrity (Figure 1). 

**Figure 2:**
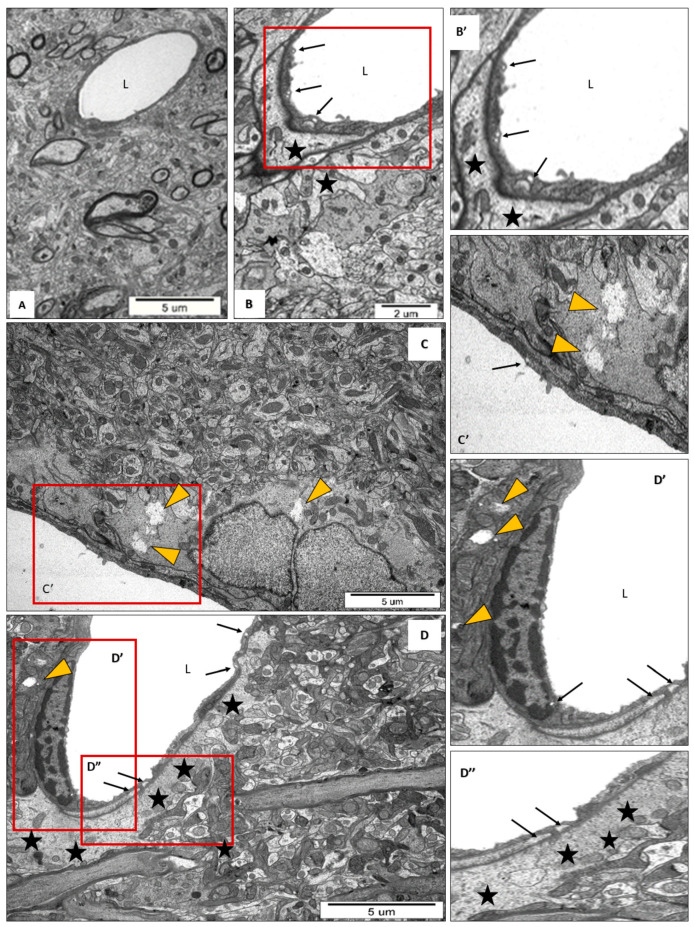
Representative TEM micrographs of brain sections taken from control (**A**) and EAE 15 d.p.i. (**B**–**D**) rats showing ultrastructural changes in capillary vessels. Pinocytotic vesicles (thin arrows), perivascular edema (asterisks), swollen areas in neuropil (yellow arrowheads). L—lumen of vessels. The images are representative for three animals per group.

Furthermore, we confirmed the increased permeability of the BBB in the brains of EAE rats by analyzing microvessels at the ultrastructural level. Features indicative of vascular leakage were observed, such as enhanced pinocytotic activity in endothelial cells, as well as local swelling of perivascular astrocytes and surrounding neuropils (Figure 2B–D). In the vicinity of some microvessels, severely edematous areas were present (Figure 2C,D). In specimens obtained from the control rat brain, normal-appearing microvessels were observed, with a proper layer of endothelium, without pinocytotic vesicles or edematous areas. Administration of DALBK significantly reduced the frequency of pathological ultrastructural changes indicative of increased vascular permeability. Edematous areas were small and sparse, and pinocytotic activity was not observed.

**Figure 3:**
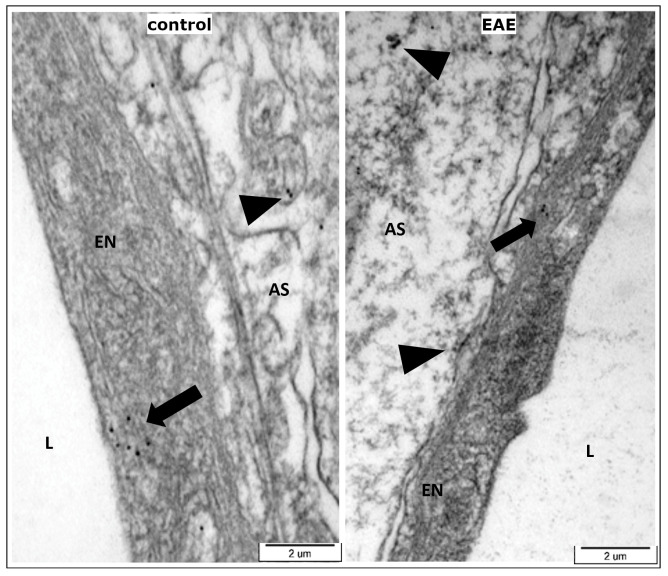
Cellular localization of the kinin B1 receptor in control and EAE 15 d.p.i. rat brain. Representative TEM micrograph showing the immunogold labelling in endothelial cells (EN; arrows) and in astroglial end-feet (AS; arrowheads); L—lumen of vessel.

In addition, vascular expression of kinin B1R was confirmed in rat brains (Figure 3). Immunogold electron microscopy revealed the presence of the reaction product in the form of electron-dense particles in endothelial cells and astroglial end-feet in brain samples obtained from both non-immunized control and EAE rats. 

### 3.3. Changes in Expression of TJ Proteins in the Brains of EAE Rats: The Effect of DALBK

**Figure 4:**
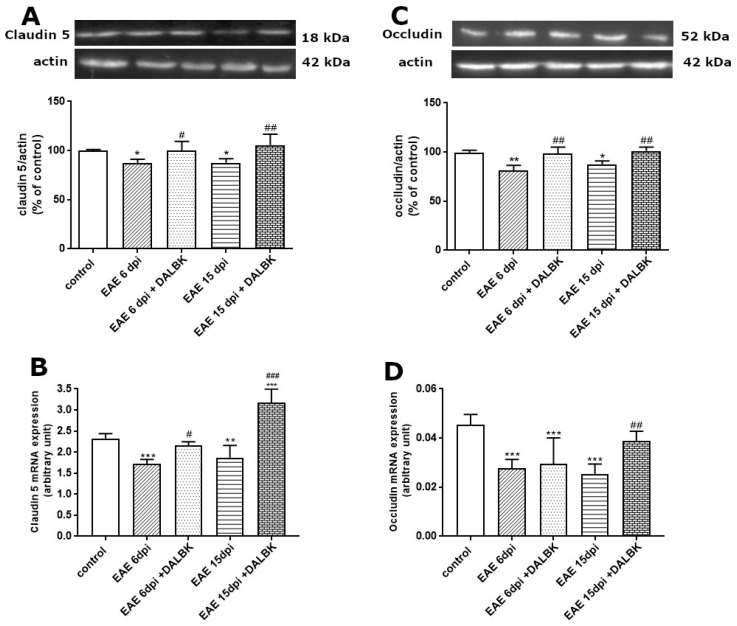
The expression of TJ proteins claudin-5 (**A**,**B**) and occludin (**C**,**D**) in brains of rats administered or not with DALBK in asymptomatic and symptomatic phases of EAE. Representative immunoblots and graphs showing the relative expression of proteins (**A**,**C**) and mRNAs (**B**,**D**), respectively. The results are means ± SD from 4 animals in each group. * *p* < 0.05; ** *p* < 0.01; *** *p* < 0.001 vs. control; ^#^ *p* < 0.05; ^##^ *p* < 0.01; ^###^ *p* < 0.001 vs. EAE (one-way ANOVA followed by post hoc Dunnett’s test).

The expression of selected TJ proteins, such as claudin-5 and occludin, was assessed in brain samples of rats from the control, EAE and EAE + DALBK groups at both the transcriptional and protein levels. The pattern of changes in relative protein levels along with the development of EAE was similar for both claudin-5 and occludin. The expression of claudin-5 decreased significantly compared to control at both 6 d.p.i. (*p* < 0.05) and 15 d.p.i. (*p* < 0.05) (Figure 4A). A significant positive effect of DALBK administration was observed, which reversed these changes in both time points. A similar profile of changes was observed at the transcriptional level, where claudin-5 mRNA decreased significantly in the early (*p* < 0.001 vs. control) and late (*p* < 0.01 vs. control) phases of EAE (Figure 4B). Administration of DALBK was found to visibly upregulate the gene, particularly in the symptomatic phase (*p* < 0.001 vs. EAE group). 

As in the case of claudin-5, the relative protein level of occludin was also reduced compared to non-immunized control rats, and DALBK reversed this effect (Figure 4C). Similarly, the level of occludin mRNA declined significantly during the course of EAE. Interestingly, in this case, the positive effect of DALBK was observed only at 15 d.p.i. (Figure 4D).

**Figure 5:**
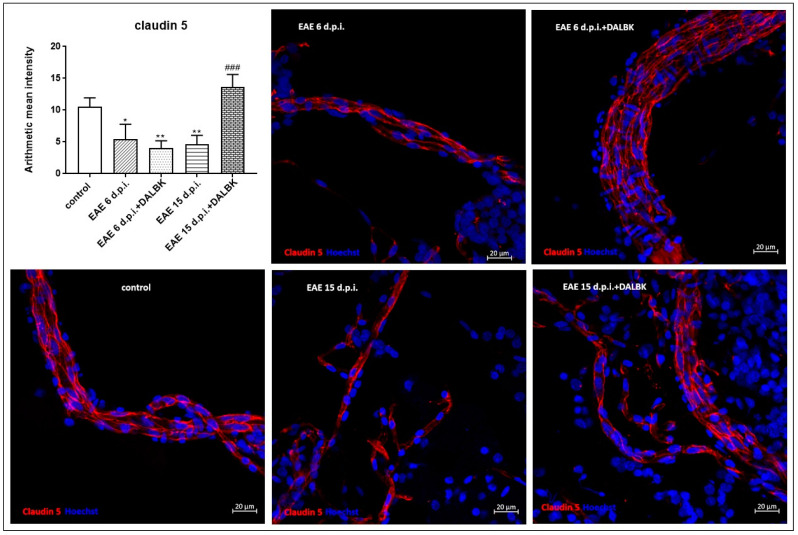
Immunostaining of claudin-5 in the capillary fractions isolated from brains of animals from control, EAE (6 d.p.i. and 15 d.p.i.) and EAE + DALBK (6 d.p.i. and 15 d.p.i.) groups. Representative confocal images of the capillary fractions labelled for claudin-5 (red) and Hoechst (blue); scale bars = 20 µm. The graph represents the mean ± SD of the fluorescence intensity measured from 9–10 sections taken from three distinct fractions in each group; * *p* < 0.05 and ** *p* < 0.01 vs. control, ^###^ *p* < 0.001 vs. EAE.

**Figure 6:**
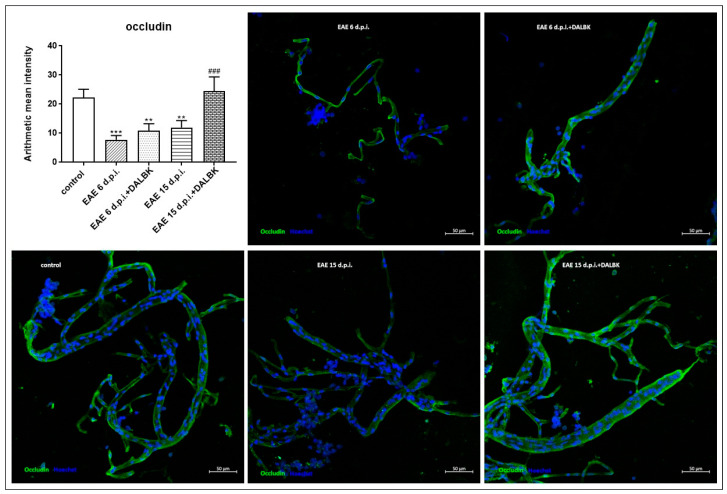
Immunostaining of occludin in the capillary fractions isolated from brains of animals from control, EAE (6 d.p.i. and 15 d.p.i.) and EAE + DALBK (6 d.p.i. and 15 d.p.i.) groups. Representative confocal images of the capillary fractions labelled for occludin (green) and Hoechst (blue); scale bars = 50 µm. The graph represents the mean ± SD of the fluorescence intensity measured from 9–10 sections taken from three distinct fractions in each group; ** *p* < 0.01 and *** *p* < 0.001 vs. control, ^###^ *p* < 0.001 vs. EAE.

Changes in the immunoreactivity of claudin-5 (Figure 5) and occludin (Figure 6) during the course of EAE were also noted in an isolated fraction of microvessels. The temporal pattern of changes expressed as the mean fluorescence intensity derived from the respective antigens was similar to that measured by the W-B method, indicating a reduced expression of both proteins. In contrast to whole brain homogenates, administration of DALBK significantly induced the expression of TJ proteins compared to the EAE group (*p* < 0.001) exclusively in the symptomatic phase (15 d.p.i.) of EAE.

### 3.4. Overexpression of GFAP in Brains of EAE Rats: The Effect of DALBK

Considering the fact that astroglia are one of the cellular components of the NVU, playing a supporting role in cerebral microvessels [6], we further investigated the temporal changes in astrocyte activation during the course of EAE. Focusing on glial fibrillary acidic protein (GFAP), which serves as the most commonly used marker to identify astrocytes and their activation state, we found overexpression of this protein. 

**Figure 7:**
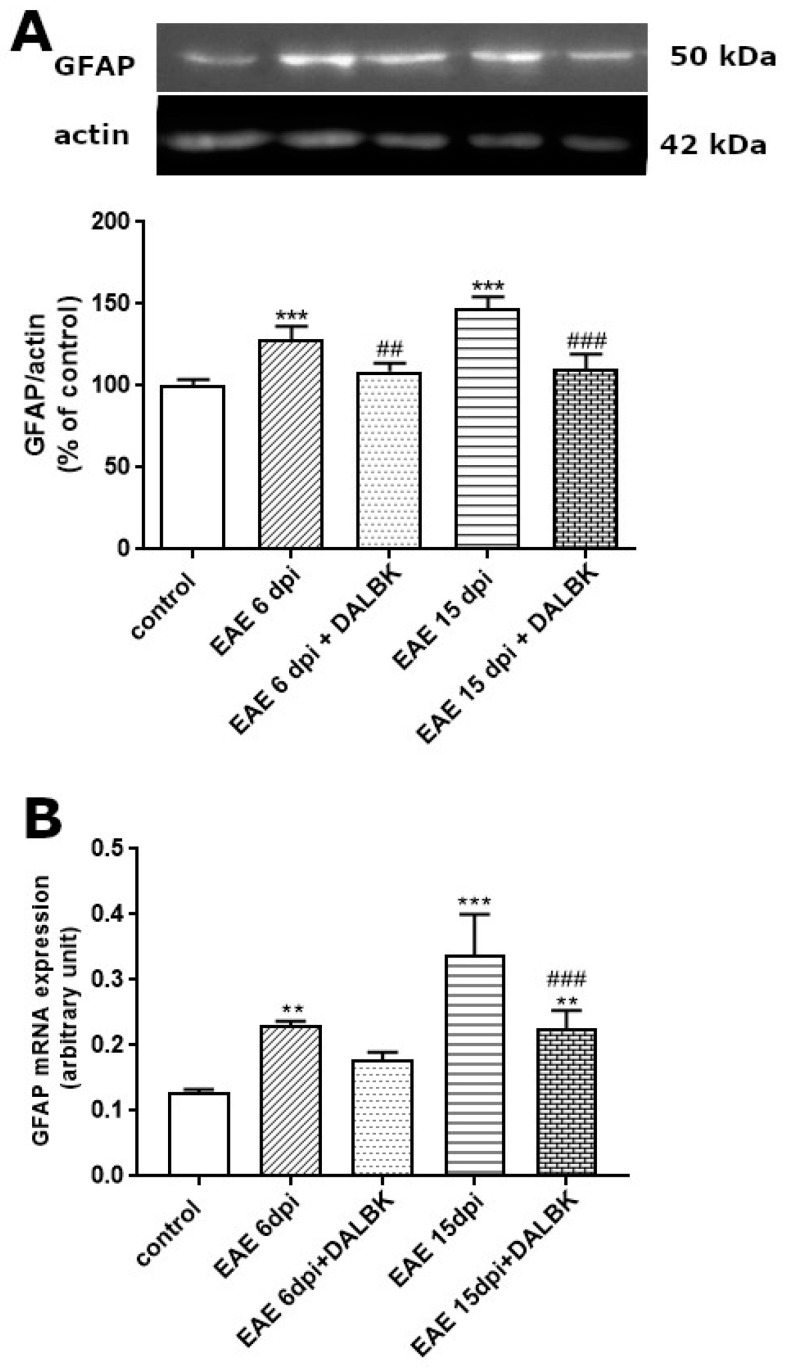
The expression of GFAP in brains of rats administered or not with DALBK in asymptomatic (6 d.p.i.) and symptomatic (15 d.p.i.) phases of EAE. Representative immunoblot and graph showing the relative expression of protein (**A**) and mRNA (**B**). The results are means ± SD from 4 animals in each group. ** *p* < 0.01; *** *p* < 0.001 vs. control; ^##^ *p* < 0.01; ^###^ *p* < 0.001 vs. EAE (one-way ANOVA followed by post hoc Dunnett’s test).

The relative concentrations of GFAP protein assessed by W-B increased significantly compared to control non-immunized rats starting from the asymptomatic phase (*p* < 0.001) and this increase was maintained in the clinical phase of the disease (*p* < 0.001) (Figure 7A).

Moreover, a similar pattern of changes in GFAP mRNA level was noted. Significant upregulation of gene was observed particularly in the clinical phase (*p* < 0.001) (Figure 7B). Administration of DALBK was found to significantly reduce overexpression of GFAP at both the protein and mRNA levels, and the reduction was evident along with the development of EAE. 

## 4. Discussion

The transvascular migration of autoreactive T cells into the CNS is a critical process that initiates the development of inflammatory lesions within the nervous tissue in both MS and EAE [23,24]. Infiltration of the CNS by immune cells is associated with increased permeability of the BBB at a very early stage of the disease and is strongly correlated with the severity of clinical symptoms [25].

In light of these data and the previously suggested involvement of B1R in the regulation of leukocyte passage via the BBB assessed in B1 R-deficient mice [15], we aimed to investigate the contribution of kinin B1R to the BBB status during the course of EAE.

The selectivity of the barrier is provided by brain endothelial cells tightly coupled by specific structures, tight junctions (TJs), which are a complex of transmembrane and cytoplasmic proteins [7]. The disruption of BBB permeability associated with local and systemic insults such as inflammation, oxidative stress and immune-related or toxic stimuli is induced by selective disruption of TJs or reduced expression of junctional proteins. Alterations in specific cytoarchitecture proteins resulting in significant changes in BBB paracellular permeability have been previously reported in experimental inflammatory pain [26], MS/EAE [27,28] and nanoparticle-induced neurotoxicity [29].

In this study, we mainly observed focal vascular alterations in the brains of EAE rats. Ultrastructural features indicative of dysfunctional microvessels were observed, such as areas of perivascular edema. In addition to vascular edema, TEM images revealed increased pinocytotic activity in endothelial cells, a feature that is associated with edema accompanying various brain pathologies [29,30].

In parallel to the ultrastructural findings, we also provide functional evidence for increased permeability of the BBB starting from the early stage of EAE (Figure 1 and Figure 2), which temporally correlates with molecular changes in tight junction (TJ) proteins, occludin and claudin-5 (Figure 4, Figure 5 and Figure 6), underlying the loss of functional integrity of microvessels. This is in line with studies showing that downregulation of claudin-5 and occludin, mediated by upregulation of astrocyte-derived vascular endothelial growth factor (VEGF), significantly contributes to BBB breakdown in an animal model of MS [31]. Indeed, overexpression of VEGF has been observed in MS plaques and in EAE lesions and has been attributed to the impairment of the BBB and upregulation of adhesion molecules [32]. Consistent with this report, which identified a mechanism of VEGF-mediated regulation of the BBB [31], are the results of our previous study showing that the protein expression of this multifunctional cytokine increases dramatically during the course of EAE starting from the asymptomatic phase of the disease (by about 400% over control; *p* < 0.01) to the onset of clinical symptoms (1300% over control; *p* < 0.001) [16]. Moreover, these findings closely correlate with the increased astroglial reactivity observed in the present (Figure 7) and previous [33] studies showing intense overexpression of GFAP in pre-clinical and clinical stages of EAE. As a key structural element of the NVU, astrocytes modulate BBB function by releasing multiple inflammatory mediators, thereby regulating migration of T cells across the BBB [34].

In this context, the markedly decreased expression of GFAP that we observed after treatment of EAE rats with DALBK (Figure 7), indicates that kinin B1 R present on astrocytes (Figure 3) may be possibly involved in the pro-inflammatory activation of astroglia. Complementary results show decreased immunoreactivity of pro-inflammatory factors (including VEGF) in the brain of DALBK-treated EAE rats [16,35].

We also provide evidence that the administration of DALBK before the onset of the clinical symptoms of EAE significantly improves BBB integrity, as assessed by reduced leakage of EB. Improved functional integrity of the BBB is correlated with molecular changes in TJ complexes. TJ proteins such as claudin-5 and occludin were upregulated in the brains of DALBK- treated EAE rats, potentially resulting in tightening of the BBB. It has been reported so far that overexpression of the tight junction protein claudin-1 in endothelial cells can promote BBB integrity in EAE rats [36].

Previous studies [15,18], including our [16], suggested that blocking B1R activity could be the basis of anti-inflammatory treatment by limiting the release of proinflammatory factors and the invasion of immune cells through cerebral vessels. Our current results indicate that attenuation of neuroinflammation by blocking B1R, which are present on both endothelial cells and perivascular astrocytes (Figure 3), is also associated with reduced activation of astroglia and with upregulation of TJ proteins, and subsequent sealing of the dysfunctional barrier at the molecular level. Astrocyte-localized B1R has already been shown to play a key role in the chronic hypersensitivity observed in the EAE rats [35], confirming the significant involvement of this specific cellular localization of the B1R in the pathomechanisms operating during EAE/MS.

In addition to improving BBB function, we also observed that treatment of EAE rats with a B1 R antagonist (DALBK) markedly, but not completely, attenuates neurological deficits and delays onset of the disease, consistent with the results previously published by Göbel et al. [15] showing that both neurological deficits and neuroinflammation are reduced in B1 R-deficient mice. The partial attenuation of neurological symptoms by DALBK indicates that B1 R-dependent mechanisms are only one of many interacting elements involved in EAE-associated neuroinflammation. However, a potential therapeutic effect of selective B1R antagonists in patients suffering from MS has been suggested [35].

In summary, we found that pharmacological inhibition of kinin B1 R not only ameliorates disease onset and clinical symptoms in EAE rats, but also reverses repairs the downregulation of TJ proteins and BBB leakage observed during the course of the disease. Blockage of B1 R in the current study was also found to significantly reduce disease-specific activation of astroglia, which may be a source of proinflammatory factors in pathological conditions. The results indicate that molecular alterations in NVU-related cellular components underlie B1 R-mediated vascular disintegration under conditions of EAE. Therefore, blocking the B1R-dependent signaling pathway may help regulate vascular permeability providing improvement in BBB function impaired during MS/EAE.

## Figures and Tables

**Table 1:** Clinical parameters of EAE rats prior to and after treatment with DALBK, an antagonist of the kinin B1 receptor, at pre-clinical (6 d.p.i.) and clinical (15 d.p.i.) stages of the disease.

Parameter	EAE		EAE + DALBK	
	6 d.p.i.	15 d.p.i.	6 d.p.i.	15 d.p.i.
Mean maximal score	0	3.90 ± 0.6	0	2.85 ± 0.2 *
Inductive phase (days)	-	6	-	4
Duration of the disease (days)	-	16 ± 1.5	-	13 ± 0.9 *
Body weight at 15 d.p.i. (g)	158 ± 0.6	139 ± 1.3	161 ± 0.9	147 ± 2.4
Number of animals	12	15	12	12

The values represent the means ± SD from the indicated number of animals; * *p* < 0.05 significantly different vs. EAE rats (one-way ANOVA with post hoc Dunnett’s test).

## Data Availability

The raw data supporting the conclusions of this article will be made available by the authors on request.
